# Tumor-derived extracellular vesicles inhibit HGF/c-Met and EGF/EGFR pathways to accelerate the radiosensitivity of nasopharyngeal carcinoma cells via microRNA-142-5p delivery

**DOI:** 10.1038/s41420-021-00794-5

**Published:** 2022-01-10

**Authors:** Changyu Zhu, Xiaolei Jiang, Hua Xiao, Jianmei Guan

**Affiliations:** 1 Personalized Drug Therapy Key Laboratory of Sichuan Province, School of Medicine, University of Electronic Science and Technology of China, Chengdu, 610072 P. R. China; 2 Department of Pharmacy, Sichuan Academy of Medical Science & Sichuan Provincial People’s Hospital, Chengdu, 610072 P. R. China; 3 Department of Pharmacy, Gansu Provincial Hospital of Traditional Chinese Medicine, Lanzhou, 730050 P. R. China; 4 Central Sterile Supply Department, Sichuan Academy of Medical Science & Sichuan Provincial People’s Hospital, Chengdu, 610072 P. R. China

**Keywords:** Cancer, Diseases

## Abstract

Radioresistance prevails as one of the largest obstacles in the clinical treatment of nasopharyngeal carcinoma (NPC). Meanwhile, tumor-derived extracellular vesicles (TEVs) possess the ability to manipulate radioresistance in NPC. However, its mechanism remains to be further explored. Therefore, the current study set out to explore the mechanism of microRNA (miR)-142-5p delivered by TEVs in regard to the radiosensitivity of NPC. Firstly, peripheral blood samples were collected from patients with radioresistance and radiosensitivity, followed by RT-qPCR detection of miR-142-5p expression. A dual-luciferase reporter assay was carried out to elucidate the targeting relationship of miR-142-5p with HGF and EGF. In addition, radiotherapy-resistant NPC cell models were established by screening NPC cells with gradient increasing radiation exposure, and co-incubated with EVs isolated from miR-142-5p mimic-transfected NPC cells, followed by overexpression of HGF and EGF. Moreover, cell viability was detected by means of MTS, cell proliferation with a colony formation assay, cell apoptosis with flow cytometry, and expression patterns of related genes with the help of Western blot analysis. NPC xenotransplantation models in nude mice were also established by subcutaneous injection of 5-8FR cells to determine apoptosis, tumorigenicity, and radiosensitivity in nude mice. It was found that miR-142-5p was poorly expressed in peripheral blood from NPC patients with radioresistance. Mechanistic experimentation illustrated that miR-142-5p inversely targeted HGF and EGF to inactivate the HGF/c-Met and EGF/EGFR pathways, respectively. NPC cell apoptosis was observed to be augmented, while their radioresistance and proliferation were restricted by EVs-miR-142-5p or HGF silencing, or EGF silencing. Furthermore, EVs-miR-142-5p inhibited growth and radioresistance and accelerated the apoptosis of radiotherapy-resistant NPC cells in nude mice by inhibiting the HGF/c-Met and EGF/EGFR pathways. Collectively, our findings indicated that TEVs might inhibit the HGF/c-Met and EGF/EGFR pathways by delivering miR-142-5p into radiotherapy-resistant NPC cells to enhance radiosensitivity in NPC.

## Introduction

Nasopharyngeal carcinoma (NPC) is an epithelial tumor originating from the nasopharyngeal mucosal lining, which is featured by obvious geographical distribution and particular prevalence in East and Southeast Asia [1]. NPC is also known to possess lymphoepithelial-like histological features and is etiologically linked to Epstein-Barr virus (EBV) infection [2]. Meanwhile, significant advancements have been made in radiotherapy techniques for the treatment of NPC, while concurrent chemotherapy is widely used for localized diseases [3]. Moreover, the overall prognosis of NPC has improved remarkably over the past decades with the advent of imaging (more accurate disease staging), radiotherapy, chemotherapy, and targeted therapies [4]. Amongst the various therapeutic approaches, intensity-modulated radiation therapy (IMRT), the current gold-standard treatment for NPC, achieves good tumor coverage and healthy tissue sparing, resulting in dose escalation and decent local control in the majority of patients [5]. However, radioresistance may cause local failure to contribute to residual or recurrent tumors in some patients, which therefore is the principal cause of NPC treatment failure [4]. Accordingly, it would be prudent to elucidate the underlying mechanism of radioresistance during NPC.

Another hot topic of interest for researchers, as heterogeneous, membrane-bound phospholipid vesicles, EVs are known to be actively released by numerous mammalian cells, including tumor cells [6]. What’s more, tumor-derived extracellular vesicles (TEVs) have been elucidated to contain various biomolecules, like metabolites, proteins, microRNAs (miRNAs/miRs), and DNAs, all of which can be transferred from cell to cell [7]. Inherently, TEVs are regarded as important components of the tumor microenvironment (TME), such that they carry out the function of communication shuttles by transducing encapsulated molecular cargos from a parent cell to a recipient cell and by directly interacting with target cells [8]. From a perspective of malignancies, a prior investigation came across the ability of TEVs to influence tumor progression of NPC via transfer of cargos (proteins, lipids, messenger RNA, miRNA, non-coding RNAs, and DNA) [9]. For instance, TEVs carrying miR-9 could repress angiogenesis in NPC [10]. On another note, EVs-shuttled miR-34c is capable of restraining malignant behavior and reversing the radioresistance of NPC cells [11]. Therefore, it would be plausible to suggest that miRNAs might play influential roles in NPC, and require further exploration in regard to radioresistance in NPC.

We focused our initial efforts on one such miRNA, namely miR-142-5p, owing to the fact that miR-142-5p inhibition promoted the resistance of lung cancer cells to gefitinib [12]. Similarly, another study documented that miR-142 downregulation was capable of accelerating NPC cell invasion and metastasis [13]. Interestingly, the TargetScan database suggests that epidermal growth factor (EGF) and hepatocyte growth factor (HGF) serve as target genes of miR-142-5p. This is particularly important as another prior study indicated that HGF was capable of facilitating cell proliferation in NPC via the HGF/c-Met pathway [14]. Meanwhile, downregulation of EGF is also known to contribute to inhibition of angiogenesis in NPC [15]. It is also noteworthy that the EGF/epidermal growth factor receptor (EGFR) pathway is regarded to assume a critical role in NPC development [16]. In this context, we asserted a hypothesis that TEVs-packaged miR-142-5p might control radioresistance in NPC via the HGF/c-Met and EGF/EGFR pathways, and subsequently set out to perform a series of experiments to verify this hypothesis, hoping to lend crucial support of further understanding in promising targets for NPC treatment.

## Results

### miR-142-5p was differentially and poorly expressed in EVs

**Fig. 1:**
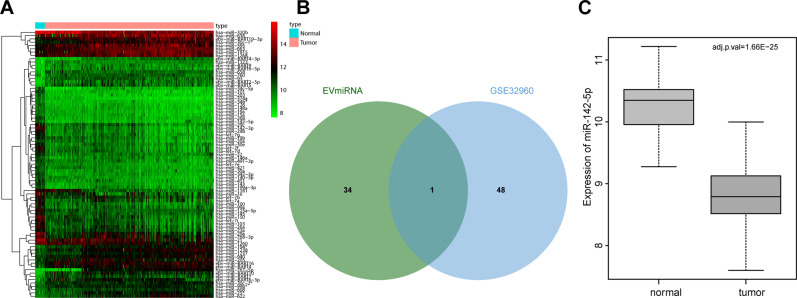
miR-142-5p is differentially expressed in EVs and poorly expressed in NPC. **A** Heat map of NPC-related miRNA microarray data (The ordinate showed the name of miRNAs, the tree on the left showed the expression clustering, and the histogram on the upper right showed the color scale). **B** The remarkably downregulated miRNA intersected with the highly expressed miRNA in peripheral blood EVs from the EVs-miRNA database. **C** Differential expression of miR-142-5p in expression dataset GSE32960 (The abscissa represented the sample type, the ordinate represented the expression value, and the upper right was the corrected difference *p-*value).

Firstly, NPC-related miRNA expression dataset GSE32960 was acquired from Gene Expression Omnibus (GEO) database and subjected to differential analyses, which revealed a total of 81 differentially expressed miRNAs in the GSE32960 dataset (Fig. 1A). In addition, the expression patterns of miRNAs in peripheral blood EVs were searched by EVs-miRNA database to obtain 35 miRNAs expressed in peripheral blood EVs. Only miR-142-5p was found at the intersection of EVs-miRNAs in peripheral blood and notably downregulated miRNAs in GSE32960 (Fig. 1B), and its expression in NPC samples was significantly downregulated (Fig. 1C). Overall, these findings revealed that miR-142-5p was differentially expressed in EVs and downregulated in NPC.

### Low expression of miR-142-5p was associated with poor prognosis in NPC patients with radioresistance

**Fig. 2:**
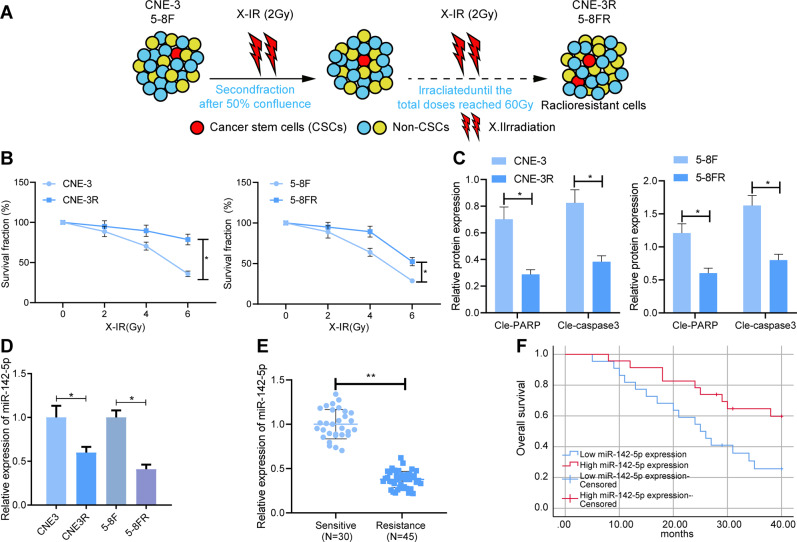
miR-142-5p downregulation is linked to radioresistance of NPC cells and poor prognosis of NPC patients. **A** The schematic diagram of constructing a cell model (5-8FR and CNE-3R) of radioresistance on human NPC cell lines 5–8 F and CNE-3. **B** The survival rate of cells at different doses of radiation. **C** The protein levels of cle-PARP and cle-caspase3 were detected by western blot after constructing a cell model (5-8FR and CNE-3R) of radioresistance. **D** RT-qPCR to measure the miR-142-5p levels in 5–8 F and CNE-3 cells, and 5-8FR and CNE-3R cells. **E** The level of miR-142-5p in peripheral blood of radiosensitive patients (*n* = 30) and radiotherapy-resistant NPC patients (*n* = 45) determined by RT-qPCR. **F** Kaplan–Meier method to analyze the relationship of miR-142-5p expression in peripheral blood and overall survival of NPC patients with radioresistance. *p < 0.05. All experiments were repeated three times.

In order to further elucidate the relationship between miR-142-5p and radioresistance, we adopted gradient increasing radiation screening to construct NPC cell models of radioresistance (5-8FR and CNE-3R, Fig. 2A). The results of colony formation assay demonstrated that 5-8FR and CNE-3R cells showed obvious radioresistance compared to their parental 5–8 F and CNE-3 cells (Fig. 2B). Meanwhile, Western blot analysis results illustrated that compared with the parental 5–8 F and CNE-3 cells, the protein expression levels of cle-caspase3 and cle-PARP in the 5-8FR and CNE-3R cells were significantly decreased (Fig. 2C and Supplementary Fig. 1A). The above results indicated that NPC radiotherapy-resistant NPC cell lines were successfully constructed. Moreover, compared with the parental 5–8 F and CNE-3 cells, miR-142-5p expression levels in radiotherapy-resistant 5-8FR and CNE-3R cells were found to be notably reduced (Fig. 2D). In addition, miR-142-5p expression levels in peripheral blood of NPC patients with radioresistance were remarkably lower than those of NPC patients with radiosensitivity (Fig. 2E). Kaplan–Meier analysis exhibited that the overall survival rate of NPC patients with high miR-142-5p expression was markedly higher than that in those with low miR-142-5p expressions (Fig. 2F). Altogether, these findings indicated that the low expression of miR-142-5p was closely related to radioresistance and poor prognosis of NPC patients.

### miR-142-5p could be transferred to radiotherapy-resistant NPC cells through EVs

**Fig. 3:**
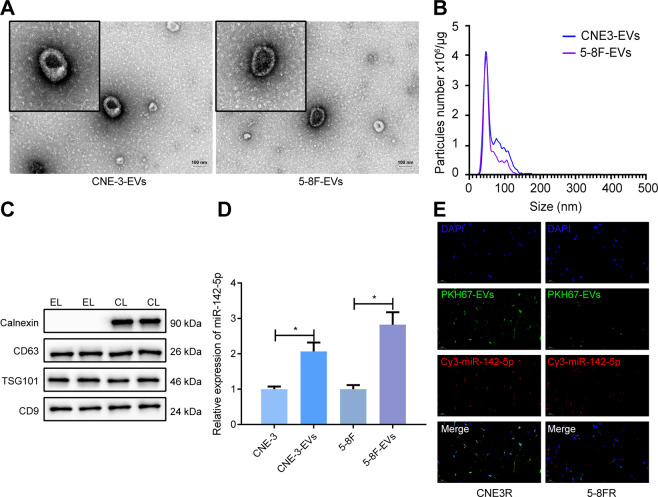
EVS derived from 5–8 F and CNE-3 cells delivers miR-142-5p into 5-8FR and CNE-3R cells. **A** The EVs of NPC cells observed by TEM (scale bar = 100 nm). **B** The size and concentration of EVs analyzed by NTA. **C** Western blot to test the EV marker proteins. **D** The expression of miR-142-5p in 5–8 F and CNE-3 cells and their EVs measured by RT-qPCR. **E** The localization of PKH67-labeled EVs and Cy3-labeled miR-142-5p in radiotherapy-resistant NPC cells observed by the confocal microscopy. The EVs were collected from parental NPC cell culture medium containing Cy3-labeled miR-142-5p and then used to culture receptor resistant NPC cells for 24 h. *p < 0.05. All experiments were repeated three times.

In order to ascertain whether TEVs influenced the radioresistance of NPC cells, EVs were isolated from the supernatants of 5–8 F and CNE-3 cells. Under transmission electron microscope (TEM), it was observed that EVs were round or oval in shape, with a complete capsule structure and were about 30–100 nm in diameter (Fig. 3A). Meanwhile, the results of nanoparticle tracking analysis (NTA) illustrated that most particles were in the range of the diameter of EVs (30–150 nm, Fig. 3B). Furthermore, the results of Western blot analysis demonstrated that EV marker proteins (CD63, Tumor Susceptibility Gene 101 [TSG101], and CD9) were present in EVs produced by NPC cells, whereas endoplasmic reticulum derived protein Calnexin was absent (Fig. 3C), and was not affected by X-IR. In a word, these findings suggested that EVs were successfully isolated from NPC cells.

Reverse transcription quantitative polymerase chain reaction (RT-qPCR) results further illustrated that miR-142-5p expression levels in 5-8F-EVs and CNE-3-EVs were significantly higher than those in the 5–8 F and CNE-3 cells (Fig. 3D). In order to determine how EVs-miR-142-5p were transferred into 5-8FR and CNE-3R cells, we adopted PKH67 to label the EVs from donor NPC cells containing Cy3-labeled miR-142-5p, and then the EVs were supplemented into the culture environment of receptor resistant NPC cells. Subsequent to 48 h, the co-localization was observed under the confocal microscope, and it was found that both red and green fluorescence were apparent in the cytoplasm of radiotherapy-resistant NPC cells, indicating that the receptor cells could absorb EVs containing miR-142-5p (Fig. 3E). Collectively, these findings indicated that EVs-miR-142-5p from 5–8 F and CNE-3 cells could be internalized by 5-8FR and CNE-3R cells.

### EVs-miR-142-5p could enhance the radiosensitivity of 5-8FR and CNE-3R cells

**Fig. 4:**
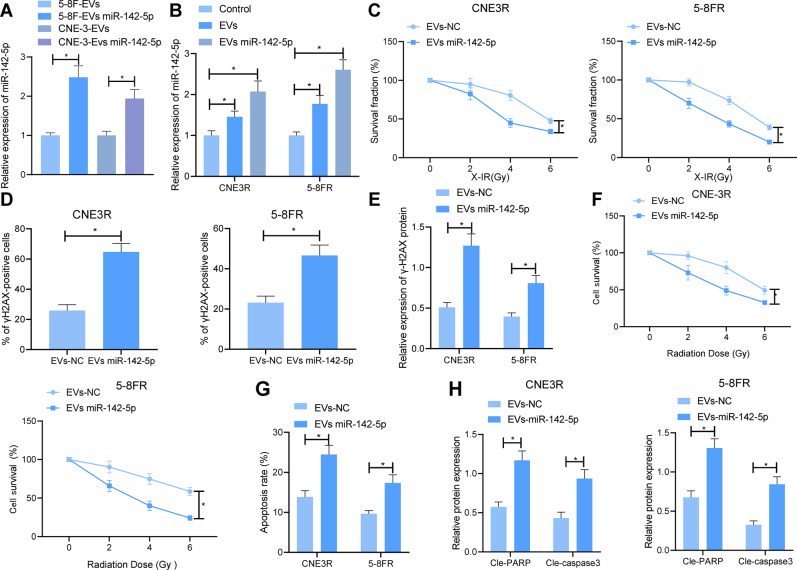
EVs-miR-142-5p facilitates NPC cell radiosensitivity to repress proliferation and increase apoptosis of NPC cells. **A** The expression of miR-142-5p in EVs secreted by 5–8 F and CNE-3 cells assessed by RT-qPCR. **B** RT-qPCR to check the expression of miR-142-5p in 5-8FR and CNE-3R cells after co-incubation with the EVs secreted by 5–8 F and CNE-3 cells. **C** Colony formation assay to detect the number of colonies formed by 5-8FR and CNE-3R cells under different doses of radiotherapy. **D** The effect of EVs-miR-142-5p on the expression of γH2AX protein in 5-8FR and CNE-3R cells detected by immunofluorescence staining. **E** Western blot to detect the effect of EVs-miR-142-5p on the expression of γH2AX protein in 5-8FR and CNE-3R cells. **F** MTS to determine the effect of EVs-miR-142-5p on the proliferation of 5-8FR and CNE-3R cells. **G** The effect of EVs-miR-142-5p on the apoptosis rate of 5-8FR and CNE-3R cells detected by flow cytometry. **H** Western blot to appraise the expression of apoptotic proteins in 5-8FR and CNE-3R cells with EVs-miR-142-5p. *p < 0.05. All experiments were repeated three times.

Furthermore, we investigated whether TEVs affected NPC cell resistance to radiotherapy by delivering miR-142-5p. miR-142-5p mimic was transfected into parental NPC cells, and then the EVs were isolated and co-incubated with the receptor cells. Subsequent results of RT-qPCR illustrated higher expression levels of miR-142-5p in the 5-8F-EVs-miR-142-5p and CNE-3-EVs-miR-142-5p compared to those in the 5-8F-EVs and CNE-3-EVs (Fig. 4A). In addition, miR-142-5p expression levels in 5-8FR and CNE-3R cells were found to be dramatically increased subsequent to co-incubation with EVs, with the highest expression in cells co-incubated with EVs-miR-142-5p (Fig. 4B). Moreover, colony formation assay displayed that the number of colonies formed by 5-8FR and CNE-3R cells was strikingly decreased by EVs-miR-142-5p, which suggested that the radiosensitivity of 5-8FR and CNE-3R cells was markedly elevated (Fig. 4C). Meanwhile, the results of immunofluorescence staining and western blot analysis demonstrated that histone H2AX on Ser 139 (γH2AX) protein expression levels in 5-8FR and CNE-3R cells treated with EVs-miR-142-5p were enhanced (Fig. 4D, E and Supplementary Fig. 1B). Together, these findings suggested that EVs-miR-142-5p could inhibit DNA damage repair in 5-8FR and CNE-3R cells.

Additionally, the results of 3-(4,5-dimethylthiazol-2-yl)-5-(3-carboxymethoxyphenyl)-2-(4-sulfophenyl)-2H-Tetrazolium (MTS) analysis illustrated that the proliferation of 5-8FR and CNE-3R cells treated with EVs-miR-142-5p was significantly decreased (Fig. 4F). Meanwhile, flow cytometry results illustrated that 5-8FR and CNE-3R cell apoptosis were markedly augmented by EVs-miR-142-5p subsequent to 6 Gy radiotherapy (Fig. 4G). As reflected by Western blot analysis results, cle-caspase3 and cle-PARP protein expression levels in 5-8FR and CNE-3R cells were facilitated by EVs-miR-142-5p (Fig. 4H). All in all, these findings indicated that EVs-miR-142-5p could enhance the radiosensitivity of 5-8FR and CNE-3R cells, thereby inhibiting proliferation and promoting apoptosis of 5-8FR and CNE-3R cells.

### miR-142-5p could target HGF and EGF to restrain the HGF/c-Met and EGF/EGFR pathways

**Fig. 5:**
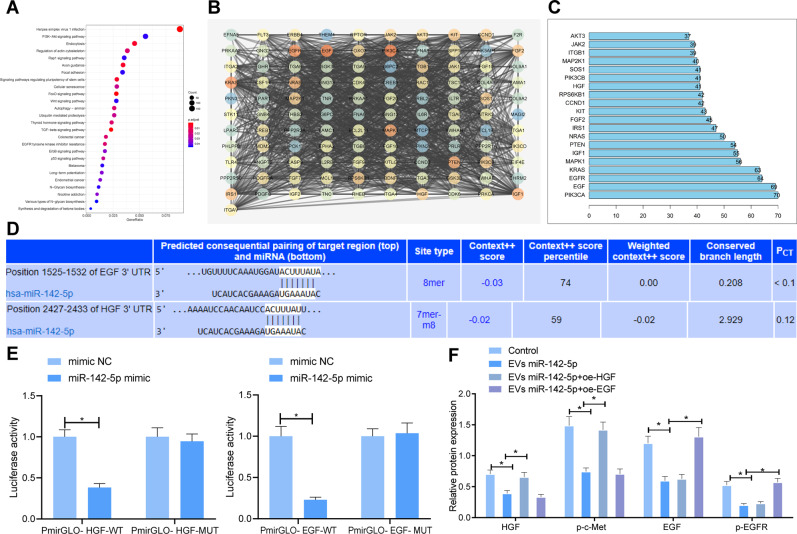
EVs-miR-142-5p blocks HGF/c-Met and EGF/EGFR pathways by targeting HGF and EGF. **A** KEGG pathway enrichment analysis of downstream target genes of miR-142-5p (The abscissa was generation, the ordinate was KEGG function item, and the histogram on the right was color scale). **B** Interaction network analysis of enriched candidate target genes in PI3K-AKT signaling pathway (Each circle in the graph represented a gene, and the lines between circles indicated that there was interaction between genes. The more interaction genes existed in a gene, the higher the degree value and the higher the core degree). **C** Statistics of degree value of core gene (Horizontal coordinate represented degree value, and ordinate represented gene name). **D** Prediction of miR-142-5p binding sites to EGF and HGF by TargetScan database. **E** Dual-luciferase reporter assay verifying the targeting relationship of miR-142-5p with HGF and EGF. **F** The protein expression of HGF, p-c-Met, EGF, and p-EGFR in 5-8FR cells lines detected by western blot. *p < 0.05. All experiments were repeated three times.

Furthermore, we predicted the target genes of miR-142-5p using the TargetScan database, and Kyoto Encyclopedia of Genes and Genomes (KEGG) pathway enrichment analysis was carried out for the candidate target genes (Fig. 5A), and it was found that the downstream target genes of miR-142-5p were primarily enriched in the PI3K-AKT pathway. Subsequently, the candidate target genes enriched in PI3K-AKT pathway were analyzed for gene interaction, and the *degree* value of core genes was calculated (Fig. 5B, C), which revealed that EGF and HGF were at the core of the whole network diagram. As a result, we speculated that EVs might affect the radiosensitivity of NPC cells by transferring miR-142-5p to regulate EGF and HGF.

Subsequently, we predicted the binding sites of miR-142-5p to HGF and EGF using the TargetScan database (Fig. 5D). The results of the dual-luciferase reporter assay manifested that the fluorescence intensity of PmirGLO-HGF-wild type (WT) and PmirGLO-EGF-WT was significantly diminished by miR-142-5p mimic, but there were no significant differences in the fluorescence intensity of PmirGLO-HGF-mutant (MUT) and PmirGLO-EGF-MUT (Fig. 5E). These findings suggested that miR-142-5p could target HGF and EGF.

Additionally, we explored whether miR-142-5p regulated the HGF/c-Met and EGF/EGFR pathways. As illustrated by the results of Western blot analysis, the protein expression levels of HGF, phosphorylation (p)-c-Met, EGF, and p-EGFR in 5-8FR and CNE-3R cells were notably decreased by EVs-miR-142-5p, while these trends were negated following over-expression of HGF or EGF (Fig. 5F). Collectively, these findings indicated that EVs-miR-142-5p could inhibit the HGF/c-Met and EGF/EGFR pathways.

### EVs-miR-142-5p enhanced radiosensitivity of radiotherapy-resistant NPC cells by targeting HGF and EGF

**Fig. 6:**
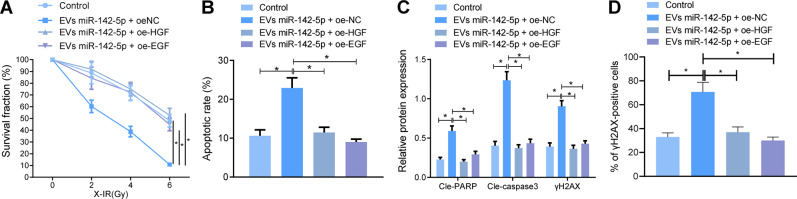
EVs-miR-142-5p enhances radiosensitivity of radiotherapy-resistant NPC cells by inactivating HGF/c-Met and EGF/EGFR pathways. **A** Colony formation assay to detect the survival rate of 5-8FR cells in each group after 6 Gy radiotherapy. **B** The apoptosis rate of 5-8FR cells detected by flow cytometry. **C** Western blot to detect the expression of PARP, Caspase-3, and γH2AX in 5-8FR cells after 6 Gy radiotherapy. **D** The expression of γH2AX protein in 5-8FR cells detected by immunofluorescence staining. *p < 0.05. All experiments were repeated three times.

We further ascertained the effect of EVs-miR-142-5p on the radiosensitivity by targeting the HGF/c-Met and EGF/EGFR pathways with the help of the 5-8FR cell line. The related genes were over-expressed in 5-8FR cells, followed by co-incubation with EVs-miR-142-5p and irradiation with 6 Gy X-IR. Subsequent results of colony formation assay and flow cytometry demonstrated that the presence of EVs-miR-142-5p, oe-EGF or oe-HGF treatment augmented 5-8FR cell survival and diminished their apoptosis rate (Fig. 6A, B). Meanwhile, Immunofluorescence staining and western blot analysis results demonstrated that in the presence of EVs-miR-142-5p, over-expression of EGF or HGF reduced cle-PARP, cle-caspase3, and γH2AX expression levels after X-IR treatment (Fig. 6C, D). In short, these findings indicated that EVs-miR-142-5p could induce apoptosis and enhance the radiosensitivity of NPC cells by disrupting the HGF/c-Met and EGF/EGFR pathways.

### EVs-miR-142-5p could enhance the radiosensitivity of 5-8FR cells in nude mice

In vitro cell studies had illustrated that EVs-miR-142-5p enhanced the radiosensitivity of NPC cells by targeting the HGF/c-Met and EGF/EGFR pathways. In order to further determine the effect of EVs-miR-142-5p on the growth and radiosensitivity of NPC cells in nude mice, we established xenotransplantation models of NPC in nude mice by subcutaneous injection of 5-8FR cells, followed by injection with normal saline, EVs, PHA-665752, or AG1478. On the 10^th^ day after modeling, 12 Gy X-IR was administered to the nude mice, and the tumor growth was detected after 25 days.

**Fig. 7:**
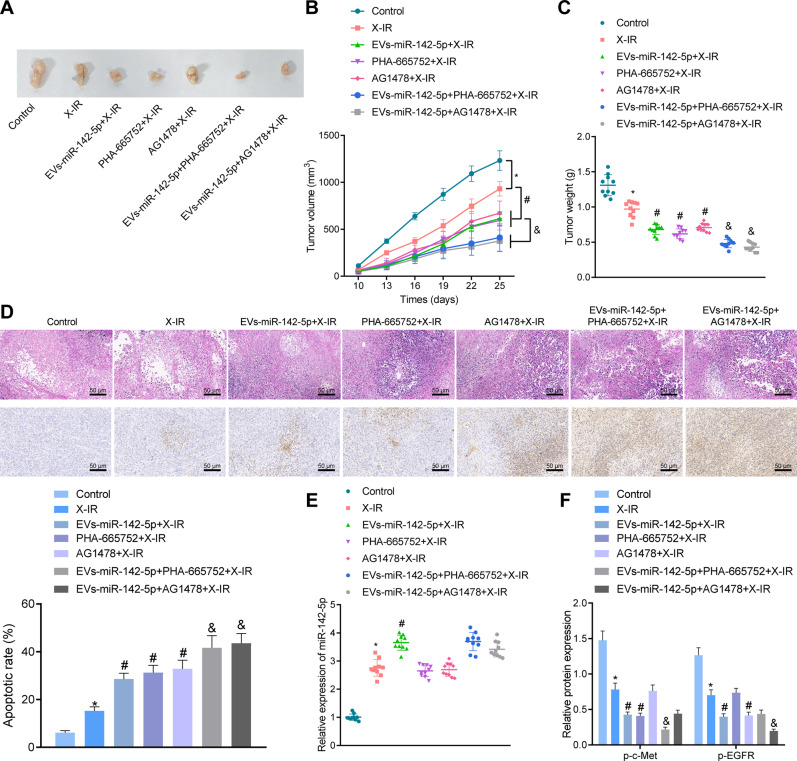
EVs-miR-142-5p could enhance the radiosensitivity of 5-8FR cells in nude mice by disrupting HGF/c-Met and EGF/EGFR pathways. **A** Naked eye observation of xenograft tumor in nude mice of each group after 20 days of 6 Gy radiotherapy. **B** Changes of xenograft tumor volume in nude mice of each group. **C** Weight changes of xenograft tumor in nude mice. **D** Representative images of H&E staining and TUNEL staining of tumors of nude mice (scale bars: 50 μm [left panel] as well as quantification of TUNEL positive cells [right panel]). E, The expression of miR-142-5p detected by RT-qPCR. **F** The protein expression of p-c-Met and p-EGFR detected by Western blot. *p < 0.05 *vs*. control mice; ^#^*p* < 0.05 *vs*. X-IR-treated mice; ^&^*p* < 0.05 *vs*. mice treated with EVs-miR-142-5p + X-IR. There were ten nude mice in each group.

It was found that X-IR treatment triggered a decline in xenograft tumor growth and weight. Moreover, the tumor size, volume, and weight were remarkably reduced by EVs-miR-142-5p in the presence of X-IR. Meanwhile, in the presence of X-IR, EVs-miR-142-5p, PHA-665752, or AG1478 were found to significantly restrain the xenograft tumor growth and weight. In the presence of EVs-miR-142-5p + X-IR, the xenograft tumor size, volume, and weight was lowered by PHA-665752 or AG1478 (Fig. 7A–C). Furthermore, the results of H&E staining and TUNEL staining illustrated that decreased tumor tissue with solid architecture, reduced large and hyperchromatic nuclei and prominent nucleoli, while strikingly enhanced percentage of apoptotic cells was observed in mice treated with X-IR. In addition, EVs-miR-142-5p, PHA-665752, or AG1478 were observed to contribute to the potent reduction of tumor tissues and enhancement of apoptotic cells in tumors in mice treated with X-IR. In the presence of EVs-miR-142-5p, the percentage of apoptotic cells was remarkably elevated by PHA-665752 or AG1478 in mice treated with X-IR (Fig. 7D). Additionally, miR-142-5p upregulation and p-c-Met and p-EGFR downregulation were observed in mice treated with X-IR. Furthermore, miR-142-5p expression levels in mice treated with X-IR were obviously augmented by EVs-miR-142-5p, while p-c-Met and p-EGFR expression levels exhibited a sharp decline. In the presence of EVs-miR-142-5p, p-c-Met expression levels were lowered by PHA-665752, whereas p-EGFR expression levels were diminished by AG1478 in mice treated with X-IR (Fig. 7E, F). Altogether, these findings indicated that EVs-miR-142-5p could inhibit the growth of xenografts and enhance the radiosensitivity and apoptosis of NPC cells in nude mice via the inhibition of HGF/c-Met and EGF/EGFR pathways.

## Discussion

Radiotherapy is lauded as the gold standard for NPC treatment, while the recent strides made in disease control and survival in NPC patients can be partly attributed to the advent of IMRT [17]. However, in addition to the damage sustained by normal tissues during radiation, radioresistance remains a serious obstacle in the successful treatment for NPC [18]. Thus, it is prudent to search for novel modalities to decrease NPC radioresistance and accelerate radiosensitivity to improve clinical outcomes in NPC patients. Meanwhile, the hard-done work of our peers has shown that tumor-derived extracellular vesicles (TEVs) play a critical role in radioresistance in different cancers [19]. In lieu of this, we set out to elucidate whether TEVs orchestrate radioresistance in NPC and subsequently performed as series of experiments to ascertain the role of TEVs in radioresistance in NPC and the potential mechanism. Overall, the obtained findings revealed that TEVs could enhance the radiosensitivity of NPC cells via the inactivation of HGF/c-Met and EGF/EGFR pathways by transferring miR-142-5p.

Firstly, our findings illustrated that miR-142-5p was poorly expressed in radiotherapy-resistant NPC patients and cells, such that overexpression of miR-142-5p resulted in diminished NPC cell radioresistance to restrain NPC cell proliferation and accelerate their apoptosis. Meanwhile, a vast number of studies have documented that various miRNAs exhibit tumor-suppressing potential in NPC. For instance, miR-142-3p was previously found to be downregulated in NPC with distant metastasis, while its upregulation led to repressed NPC cell invasion and metastasis [13]. In addition, a prior study came across decreased expressions of miR-590-3p and miR-1275 in NPC tissues, while their over-expression depressed the NPC cell proliferative, migrating, invasive properties [20]. Moreover, another miRNA, namely miR-99a, was previously illustrated to be downregulated in NPC cells and further associated with increased NPC cell proliferation [21]. It is also noteworthy that miR-142-5p has been documented to serve as an anti-oncogene in several cancers. Zhu et al. observed that miR-142-5p was lowly expressed in pancreatic cancer tissues and cell lines, while ectopic overexpression of miR-142-5p could reduce pancreatic cancer cell proliferation [22]. Besides, the study performed by Li et al. indicated that miR-142-5p facilitated cisplatin-caused ovarian cancer cell apoptosis *via* numerous anti-apoptotic genes [23]. Similarly, miR-142-5p is known to be capable of restraining resistance to gefitinib in lung cancer cells [12]. Altogether, these findings and evidence make it plausible to highlight miR-142-5p as a repressor of radioresistance in NPC.

Additionally, further experimentation in our study manifested that miR-142-5p could be delivered into radiotherapy-resistant NPC cells by means of TEVs. There is substantial evidence that highlights the significance of miRNAs loaded in TEVs and their subsequent roles as orchestrators in intercellular communication, either in the TEM or at distant sites [24]. More importantly, we uncovered that TEVs carrying miR-142-5p augmented NPC cell radiosensitivity, which resulted in curtailing cell proliferation and accelerating cell apoptosis. It is also known that TEVs possess the ability to influence the TEM and further manipulate cancer developments that may result in drug resistance and cancer recurrence [25]. Moreover, several TEVs carrying miRNAs have been previously recognized to confer pivotal roles in drug resistance of cancers [26]. For example, EVs upregulating miR-34c resulted in the suppression of malignant behavior and radioresistance of NPC cells [11]. Together, these findings indirectly support the tumor-suppressing function of TEVs-delivered miR-142-5p in radioresistance in NPC.

Furthermore, miRNAs are capable of post-transcriptionally orchestrating expression of target genes by binding to their 3'untranslated region (UTR) [27]. In this context, our findings illustrated that TEVs-delivered miR-142-5p targeted HGF and EGF to disrupt the HGF/c-Met and EGF/EGFR pathways, ultimately diminishing NPC cell radioresistance to inhibit cell proliferation and facilitate cell apoptosis. Corroborating findings have been reported in prior studies, wherein HGF upregulation was found to bring about the repression of NPC cell apoptosis and promotion of their proliferation [28]. It should also be noted that a previous study discovered that ectopic expression of HGF facilitated NPC cell proliferation by activating the HGF/c-Met pathway, which is in much accordance with our findings [14]. In addition, the investigation performed by Zhu et al. indicated that blockade of the HGF/c-Met pathway would result in restrained radioresistance and augmented cell apoptosis in human non-small-cell lung cancer [29]. Moreover, another prior study illustrated that EGF silencing sensitized NPC cells to radiotherapy [30]. Similarly, EGFR downregulation was previously shown to accelerate the radiosensitivity of NPC cells [31].

**Fig. 8:**
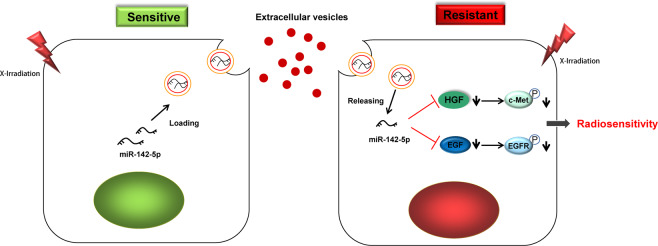
Mechanism of miR-142-5p delivered by TEVs in radiosensitivity of NPC by regulating HGF/c-Met and EGF/EGFR axis.

In summary, findings obtained in our study supported the notion that TEV-packaged miR-142-5p could enhance the radiosensitivity of NPC cells by blocking the HGF/c-Met and EGF/EGFR pathways (Fig. 8). Our study provides a new theoretical basis and molecular target for improving NPC radioresistance. However, more efforts are expected in the clinical application of targeted therapy in the future.

## Materials and methods

### Bioinformatics analysis

Firstly, NPC-related miRNA expression dataset GSE32960 comprising of 18 normal control samples and 312 NPC samples was retrieved from the GEO database, and subjected to differential analysis using the R language “limma” package. The product *p-*value was corrected with the false discovery rate (FDR) method, with *|* log FC | *>* 1 and *adj. p-*value < *0.05* were used as the screening criteria for differentially expressed miRNAs in NPC. In addition, miRNAs with expression above 2000 in EVs from tumor cells were also searched using the EVs-miRNA database. The downstream target genes of miR-142-5p were further predicted using the TargetScan database. Next, the R language “clusterProfiler” package was adopted to analyze the KEGG pathway enrichment of the predicted target genes. Gene interaction analysis of candidate target genes was then performed using the STRING database. The gene interaction network was constructed with the help of the Cytoscape v3.7.1 software, and the degree value was counted. Afterward, the binding site information between miR-142-5p and the target genes was obtained from the TargetScan database.

### Clinical samples

A total of 75 NPC patients from May 2016 to May 2017 at the Sichuan Academy of Medical Science & Sichuan Provincial People’s Hospital were enrolled in the current study, including 45 patients with radioresistance and 30 patients with radiosensitivity. The clinicopathological features of the patients are shown in Supplementary Table 1. The inclusion criteria for radioresistance were as follows [32]: the presence of residual tumors and/or lymph node in the follow-up 3 months after radical radiotherapy; occurrence of pharyngeal and/or new lymph node enlargement within 1 year after radical treatment; recurrence occurred within 1–2 years after the radiological examination. The inclusion criteria for radiosensitivity were as follows [32]: absence of residual tumor and/or lymph nodes in the primary tumor during the follow-up 3 months after radical radiotherapy; no nasopharynx and/or new lymph node enlargement within 1 year after radiotherapy; no recurrence or metastasis within 1–2 years after the radiological examination. All included patients were followed up for more than 3 years without recurrence or metastasis.

### Cell culture

Two different types of human NPC cell lines (5–8 F and CNE-3, verified to be free of pollution) and human embryonic kidney cell line HEK293T were procured from Procell (Wuhan, Hubei, China) for in vitro cell experimentation. The 5–8 F and CNE-3 cells were cultured in Roswell Park Memorial Institute 1640 medium (Gibco, Carlsbad, California) encompassing 10% fetal bovine serum (FBS). Meanwhile, the HEK293T cells were cultured in Dulbecco’s Modified Eagle Medium (DMEM, HyClone Company, Logan, UT) encompassing 10% FBS (16140071, Thermo Fisher Scientific, Waltham, Massachusetts).

### Construction of radiotherapy-resistant NPC cell lines

The 5–8 F and CNE-3 cells were seeded in T25 flasks at a density of 1 × 10^5^, and then cultured in DMEM (Invitrogen, Carlsbad, CA) containing 10% FBS and 1% penicillin–streptomycin. After 24 h of culture, the cells were irradiated with a linear accelerator (2100EX, Varian Medical Systems, Palo Alto, Calif) at a rate of 300 Gy/min at room temperature. After 5–8 F and CNE-3 cells were treated with a sub-lethal dose of radiation (13 Gy), the surviving cells were selected and cultured to produce the primary passage of sub-clonal cells. Sub-cloned cells were subjected to sub-lethal doses of radiation another time, and the surviving cells were selected to produce the next passage of sub-cloned cells. Cells at the fourth passage were regarded as the radiotherapy-resistant sub-clonal cell lines, named 5-8FR and CNE-3R, respectively. The 5-8FR and CNE-3R cells in 4–10 passages were tested after the termination of radiotherapy. Afterward, all the aforementioned cells were cultured in a humidified incubator at 37 °C with 5% CO_2_ in the air.

### Cell transfection and lentivirus infection

As per the instruction manual of Lipofectamine transfection kits (Invitrogen), logarithmically growing cells were seeded in a 6-well plate at a density of 6.0 × 10^5^ cells/well. Subsequently, miR-142-5p mimic and inhibitor were transfected into the 5-8FR and CNE-3R cells. Briefly, each well was added with 25 pmol mimic and inhibitor and 10 μL transfection reagents, with a final concentration of 10 pmol/mL. The cells were then gently shaken and cultured at 37 °C with 5% CO_2_ in air. In order to investigate the effect of HGF and EGF on radiosensitivity of NPC cells, 5-8FR and CNE-3R cells were further transfected with 100 nM pcDNA3.1-HGF, pcDNA3.1-EGF or their negative control (NC) (GenePharma, Shanghai, China) respectively. Each experimental group was repeated three times independently. The cells were continuously cultured for 48 h and then collected for subsequent experimentation.

In order to obtain EVs from 5–8 F and CNE-3 stably over0expressing miR-142-5p, the 5–8 F and CNE-3 cells were infected with lentivirus. Briefly, the 293 T cells were transfected with 3 μg pLenti-miR-142-5p, 1 μg pCMV-VSV-G, and 3 μg pCMV-Delta8.9 using Lipofectamine 3000 reagent (Invitrogen). After 20 h, the medium was replaced with medium containing 5% FBS. After about 48 h, the supernatant containing the virus was filtered through a 0.45 μm cellulose acetate filter (Millipore, Billerica, MA), and stored at −80 °C. In order to construct the cell line expressing miR-142-5p, the supernatant containing virus was diluted three times with serum-free DMEM containing 10 μg/mL polyamine (Yeasen, Shanghai, China). Next, the target cells with a density of 40% were incubated with the mixture containing virus for 8 h, and then the mixture was renewed with DMEM containing 10% FBS. Finally, the cells were screened with 10 μg/mL puromycin (Sigma-Aldrich, St. Louis, MO) for 4 weeks.

### Isolation and purification of EVs

The FBS was pre-centrifuged at 100,000 × g for 18 h to remove the EVs in serum. After cell confluence reached about 80%, the supernatant was removed. Next, the medium was replaced with 10% FBS culture medium without EVs, and cultured in a humidified incubator at 37 °C with 5% CO_2_ in air for 48 h. Subsequent to the collection of 5–8 F and CNE-3 cell suspension, the EVs were extracted from 5–8 F and CNE-3 cells by means of ultracentrifugation. All the centrifugation steps were completed at 4 °C, and the other steps were performed on ice. The specific process was as follows: cell debris and dead cells were removed by centrifugation at 500 × g for 10 min and at 2000 × g for 20 min using a cryogenic centrifuge (Eppendorf, Hamburg, Germany). The supernatant was then centrifuged at 10,000 × g for 45 min to remove the cell debris and large EVs using a high-speed centrifuge (Beckman Coulter Life Sciences, Brea, CA). Afterward, the supernatant was filtered using a 0.22 μm filter, and the EVs were extracted with an ultrahigh-speed centrifuge at 110,000 × g for 90 min. The precipitates were centrifuged again with phosphate buffer saline (PBS) suspension. Finally, the precipitate was suspended in 50–100 μL PBS and stored at −80 °C.

### TEM

Suspensions of EVs isolated from 5–8 F and CNE-3 cell were negatively stained with osmium tetroxide, phosphotungstic acid, ammonium molybdate, and acetic acid using the floating method, respectively. Next, the EVs samples were observed by means of TEM (H-7650, HITACHI, Tokyo, Japan) under an accelerated voltage of 80 kV.

### Particle size analysis of Nanosight EVs

NTA measurements were performed using a Nanosight NS3000 system (Nanosight, Amesbury, UK) equipped with a blue laser (405 nm). The EVs were irradiated with the laser, and their motion in the Brownian motion was recorded in 9 s of sample video, and subsequently analyzed using the NTA analysis software (version 2.3, Nanosight). Capture settings and analysis settings were performed manually as per the manufacturer’s protocols.

### Western blot analysis

The cultured 5–8 F and CNE-3 cells were detached by trypsin and lysed with an enhanced Radio-Immunoprecipitation assay cell lysis buffer containing protease inhibitor (BOSTER, Wuhan, Hubei, China). Next, the EV precipitates separated from the same amount of medium (10 mL) were lysed in 200 μL lysis buffer (Roche Diagnostics GmbH, Mannheim, Germany). Afterward, the protein concentration was determined with a bicinchoninic acid protein quantitative kit (BOSTER). The proteins were then separated by 10% sodium dodecyl sulfate–polyacrylamide gel electrophoresis and transferred onto a polyvinylidene fluoride membrane. Subsequently, the membrane was sealed at room temperature for 2 h with 5% bovine serum albumin to block nonspecific binding, and then probed overnight with diluted primary antibodies (Abcam, Cambridge, UK) to cle-poly(ADP-ribose) polymerase (PARP) (ab32064, dilution ratio of 1:1000), cle-caspase3 (ab32042, dilution ratio of 1:500), γH2AX (ab124781, dilution ratio of 1:500), p-c-Met (ab68141, dilution ratio of 1:1000), HGF (ab178395, dilution ratio of 1:500), EGF (ab206423, dilution ratio of 1:500), p-EGFR (ab40815, dilution ratio of 1:500), β-actin (ab8226, dilution ratio of 1:1000), CD63 (ab134045, dilution ratio of 1:1000), CD9 (ab223052, dilution ratio of 1:1000), TSG101 (ab125011, dilution ratio of 1:1000), and Calnexin (ab133615, dilution ratio of 1:1000) at 4 °C. Afterward, the membrane was re-probed for 1 h with horseradish peroxidase-tagged goat anti-rabbit (ab205719, dilution ratio of 1:2000) or goat anti-mouse (ab6808, dilution ratio of 1:2000) secondary antibodies (Abcam). Later, the membrane was developed by electro-generated chemiluminescence (EMD Millipore Corporation, Billerica, MA) at room temperature for 1 min, and then the excess ECL reagent was removed. After that, the membrane was sealed with preservative films, exposed with the X-ray films in the cassette for 5–10 min before developing and fixing. The Image J analysis software was adopted to quantify the gray level of each band in Western blot images. β-actin was regarded as a normalizer for cell lysate proteins and Ponceau red as a normalizer for EV marker proteins.

### RT-qPCR

The Trizol reagent (16096020, Thermo Fisher Scientific) was employed for extracting the total RNA content from tissues and cells. cDNA of miRNA was synthesized from the total RNA of cells and tissues using the miRcute Plus miRNA First-Strand cDNA Synthesis Kits (TIANGEN Biotechnology Co. Ltd, Beijing, China). Next, the synthesized exogenous reference cel-miR-39 (1 pmol per sample, TIANGEN Biotechnology Co. Ltd) was added to 350 μL medium or 100 μg EVs in advance. The miRNA was extracted from these samples with a mirVana PARIS kit (Ambion Company, Austin, TX). Real-time PCR of miRNA was carried out using a miRcute Plus miRNA qPCR Kit (TIANGEN Biotechnology Co. Ltd). In cell and tissue lysates, miRNA expression was standardized to U6. Meanwhile, miRNA expression in the medium and EVs were standardized relative to the exogenous reference cel-miR-39. miRNA universal reverse primers were provided by the miRcute Plus miRNA First-Strand cDNA Synthesis kit. The remaining primers were synthesized by Sangon (Shanghai, China). The primers are manifested in Supplementary Table 2. The 2^−ΔΔCT^ method was employed for analysis.

### Immunofluorescence staining

In accordance with the manufacturer’s instructions (Mirus Bio Corporation, Madison, WI), pre-miR miRNA precursors (hsa-miR-142-5p, Ambion Company) were labeled with a Label IT siRNA Tracker Cy3 kit. HiPerFect (Qiagen Company, Hilden, Germany) was adopted to transfect the 5-8FR and CNE-1R cells with 100 nM Cy3-labeled pre-miR miRNA precursor. After one day of incubation, the medium was employed for EV preparation.

The green lipophilic fluorescent dye PKH67 (Sigma-Aldrich) was adopted to label the EVs including Cy3-miR-142-5p. PKH67-labeled EVs including Cy3-miR-142-5p were cultivated with cultured 5-8FR and CNE-3R cells. Following incubation, the 5-8FR and CNE-3R cells were observed under a confocal microscope (FV10i, Olympus, Tokyo, Japan). The 4',6-Diamidino-2-Phenylindole (Abbott Laboratories, Chicago, IL) was applied for nuclear staining.

### Colony formation experiment

The density of 5-8FR and CNE-3R cell suspension was adjusted to 1 × 10^4^ cells/mL. Next, the cells were seeded in triplicate in a 6-well plate. After 24 h, the cells were exposed to different doses of X-irradiation (IR) (2, 4, or 6 Gy). Afterward, the cells were cultured at 37 °C for 9–12 days until visible colonies were formed. The colonies were then stained with crystal violet and counted. The survival fraction was calculated as (number of colonies/number of seeded cells)/(number of non-irradiated colonies/number of seeded cells).

### DNA damage detection

The 5-8FR and CNE-3R cells at a density of 1 × 10^5^ were seeded in a 6-well plate with cover glass on each well. Next, the 5-8FR and CNE-3R cells were cultured for 24 h and treated with 4-Gy IR. Following IR treatment, 5-8FR and CNE-3R cells were fixed with 4% paraformaldehyde at 4 °C for 15 min. After that, the cells were ruptured with 0.2% TrtorX 100 for 15 min and sealed with goat serum working solution for 1.5 h. Afterward, the cells were incubated with γ-H2AX primary antibody (dilution ratio of 1:1000) for 2 h and then incubated with labeled secondary antibody (dilution ratio of 1:500) for 1 h. Later, the cover glass was sealed with 90% glycerin, and the number of green γ-H2AX focus was counted using a fluorescence microscope.

### Flow cytometry

After 12 days of 6 Gy radiotherapy, Annexin V-fluorescein isothiocyanate/propidium iodide double staining (556547, Becton, Dickinson and Company, NJ) was adopted to detect the apoptosis of 5-8FR and CNE-3R cells. Apoptosis of 5-8FR and CNE-3R cells was detected with the help of a flow cytometer (Becton, Dickinson and Company) at 488 nm.

### Dual-luciferase reporter assay

The WT and MUT reporter plasmids (PmirGLO-HGF-WT, PmirGLO-HGF-MUT, PmirGLO-EGF-WT, or PmirGLO-EGF-MUT) containing binding sites in the 3'UTR of HGF and EGF were designed and provided by GenePharma. The mimic NC and miR-142-5p mimic were co-transfected with PmirGLO-HGF-WT, PmirGLO-HGF-MUT, PmirGLO-EGF-WT, or PmirGLO-EGF-MUT into HEK293T cells, respectively, and the cells were collected after 48 h. Changes in luciferase activity were detected following the operation method of the dual-luciferase detection kit (D0010, Solabio, Beijing, China) on a GLomax20/20 Luminometer fluorescence detector (E5311, Shaanxi Zhongmei Biotechnology Co., Ltd., Shaanxi, China).

### Xenograft tumor model in nude mice

A total of 70 BALB/c nude mice were purchased from Shanghai Experimental Animal Center, Chinese Academy of Sciences (Shanghai, China). Next, 2 × 10^6^ logarithmically growing 5-8FR cells were resuspended in 200 μL DMEM and injected subcutaneously into the nude mice. When the tumor diameter was about 5 mm, a single dose of 12 Gy X-IR (600 cGy/min was used for vertical irradiation at room temperature for 1 min) was utilized to irradiate the tumor. Afterward, the nude mice were injected with 500 μL normal saline via a caudal vein, irradiated with 12 Gy X-IR at 10^th^ day, injected with EVs-miR-142-5p (100 μg EV precipitate was dissolved in 500 μL PBS) via tail vein and then irradiated with 12 Gy X-IR at 10^th^ day, injected with 10 μmoL/L c-Met specific inhibitor PHA-665752 via tail vein and irradiated with 12 Gy X-IR at 10^th^ day, injected with 10 μmoL/L EGFR specific inhibitor AG1478 via the tail vein and irradiated with 12 Gy X-IR at 10^th^ day, injected with EVs-miR-142-5p + 10 μmoL/L PHA-665752 via the tail vein and irradiated with 12 Gy X-IR at 10^th^ day, and EVs-miR-142-5p + 10 μmoL/L AG1478 via the tail vein and irradiated with 12 Gy X-IR at 10^th^ day (ten mice/group). Afterward, the tumor size was measured every 2 days after tumor formation. The tumor volume was calculated according to the following formula: tumor volume (mm^3^) = 1/2 × length × (width)^2^. The tumor was removed after 25 days of tumor formation, and the size and weight of tumor were compared. Mouse tumor tissues were fixed in 10% formalin for 24 h before routine dehydration and paraffin embedding. Tumor tissue samples were sectioned at a thickness of 3 μm and stained with hematoxylin-eosin (H&E) using standard methods, followed by microscopic photographing (at ×200 magnification).

### Terminal deoxy(d)-UTP nick end labeling (TUNEL) assay

Paraffin sections of mouse tumor tissues were deparaffinized by two 5-min immersions with xylene and one 3-min immersion with gradient ethanol (100, 95, 90, 80, and 70%) each. Next, the sections were treated with Proteinase K working solution (20 μL/mL) for 15–30 min at 21–37 °C or permeabilized for 8 min, and then reacted with PBS containing 2% hydrogen peroxide for 5 min at room temperature. Subsequent to the preparation of the TUNEL reaction mixture, the treatment groups were mixed completely with 50 μL TdT + 450 μL fluorescein-labeled dUTP. Afterward, the negative control group was added with 50 μL fluorescein-labeled dUTP solution. The positive control group was firstly reacted with 100 μL DNase 1 with the same latter steps as the treatment group. After the slides were dried, the excess liquid around the sections was carefully blotted off using filter paper, and a 50 μL TUNEL reaction mixture was added on the specimen for 1-h reaction in conditions void of light at 37 °C. The sections were incubated with pre-warmed stop reaction buffer for 30 min at 37 °C. The apoptotic cells were counted under a fluorescence microscope (excitation wavelength was 450–500 nm, and detection wavelength was 515–565 nm) following the addition of one drop of PBS. Next, the sections were reacted with 50–100 μL diaminobenzidine substrates at 15–25 °C for 10 min. Subsequent to photographing, the sections were counterstained with hematoxylin or methyl green. Following gradient alcohol dehydration, xylene clearing, and neutral gum mounting, the sections were supplemented with a drop of PBS or glycerol, and the apoptotic cells were observed and photographed under an optical microscope (a total of 200–500 cells).

### Statistical analysis

Statistical analyses were performed using the SPSS 21.0 software (IBM Corp. Armonk, NY). Measurement data were presented as mean ± standard deviation. An unpaired *t*-test was adopted for comparisons between two groups. One-way analysis of variance (ANOVA) was applied for comparisons among multiple groups, while repeated measurement ANOVA was used to compare tumor data at different time points, followed by Bonferroni post-hoc test. Cell survival data at different time points were compared by two-way ANOVA. The survival rate of NPC patients was calculated using the Kaplan–Meier method. Log-rank test was adopted for univariate analysis. A value of *p* < 0. 05 was regarded to be statistically significant.

## Supplementary information

Supplementary Information

## Data Availability

The datasets generated and/or analyzed during the current study are available from the corresponding author on reasonable request.
