# Supplementary material for: Tumor-derived extracellular vesicles inhibit HGF/c-Met and EGF/EGFR pathways to accelerate the radiosensitivity of nasopharyngeal carcinoma cells via microRNA-142-5p delivery
Source: Cell Death Discov. 2022 Jan 10;8:17. doi: 10.1038/s41420-021-00794-5 (PMC8748649; doi:10.1038/s41420-021-00794-5)
Supplement: Supplementary file 1 — Supplementary Information [file 41420_2021_794_MOESM1_ESM.docx]

**Supplementary Information**

**Supplementary Figure 1** A, Representative protein bands of Figure 2C. B, Representative protein bands of Figure 4E.

**
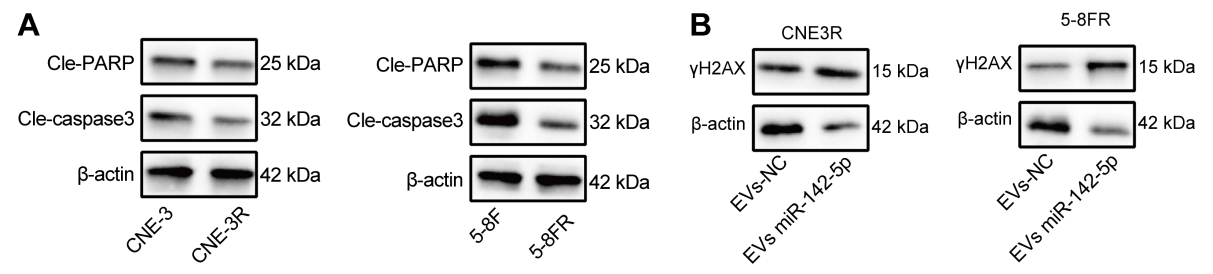
**

**Supplementary Table 1** Clinicopathological features of 75 NPC patients

| Features | Radiosensitivity group (N = 30) | Radioresistance group (N = 45) |
| --- | --- | --- |
| Gender |  |  |
| Man | 18 | 30 |
| Woman | 12 | 15 |
| Age | 43.75 ± 8.08 | 46.18 ± 9.35 |
| Clinical stages |  |  |
| I | 5 | 5 |
| II | 12 | 12 |
| III | 10 | 21 |
| IV | 3 | 7 |
| Tissue typing |  |  |
| Keratinizing squamous cell carcinoma | 11 | 16 |
| Non-keratinizing carcinoma | 19 | 29 |
| Differentiation type |  |  |
| Differentiated type | 17 | 19 |
| Undifferentiated type | 13 | 26 |

**Supplementary Table 2** Primers for RT-qPCR

| Names | Sequences (5′-3′) |
| --- | --- |
| miR-142-5p | F: CAUAAAGUAGAAAGCACUACU |
| cel-miR-39 | F: CACCGGGTGTAAATCAGCTTG |
| Human U6 snRNA | F: CTCGCTTCGGCAGAC |
